# Strategies for transnational projection through international book fairs

**DOI:** 10.1007/s11059-023-00690-0

**Published:** 2023-06-15

**Authors:** M. Carmen Villarino Pardo

**Affiliations:** Grupo Galabra-Universidade de Santiago de Compostela, Santiago de Compostela, Spain

**Keywords:** International book fairs, Soft power, Guest of honor, Transnational projection, Culture

## Abstract

The following article aims to analyze the projection and representation of literary systems in some of the main international book fairs (IBF) at present, based on theoretical-methodological proposals from the Sociology of Literature and Culture (Bourdieu in ARSS 145, 3–8, 2002; Sapiro Translatio. Le marché de la traduction en France à l’heure de la mondialisation, CNRS Éditions, 2008; Even-Zohar, Polisistemas de Cultura. Laboratorio de investigación de la cultura, 2017). We begin with the hypothesis of considering IBFs as places for the acquisition of capital (literary, cultural, economic and symbolic) and for negotiating struggles for positions of greater or lesser centrality in the transnational literary field and in the world publishing market, and in terms of the image of countries, cultures, or cities. In those IBFs offering the position of Guest of Honor, this becomes a privileged opportunity—for governments, institutions and different actors involved in the book chain—for the international projection of the image of a country, culture, city or literary system. Different strategies for cultural and literary promotion in this worldwide scenario of symbolic goods will be discussed, with special attention to the case of Brazil (contrasted, partly, with that of Spain).

## International book fairs

The recent challenges arising from globalization and internationalization phenomena and the increasing specific weight—also quantifiable at various levels (Carrasco Arroyo, 2006)—that culture has come to have in these processes, reveal a (growing) interest shared by many countries and cities in matters of innovation, institutional strengthening, entrepreneurship, cultural diversity, and sustainable development.

Linked to this are the cultural and literary events we call festivals/celebrations and book fairs. These are activities in which the literary, cultural, and economic spheres converge in spaces—usually urban—that seek singularization and projection strategies. Academic interest in their analysis has increased significantly in the current century.^1^ (Driscoll & Squires, 2020; Flood, 2007; Giorgi et al., 2013; Martínez Tejero, 2019; Sapiro, 2016; Villarino Pardo, 2022).

In this article we will focus on International book fairs (IBF). In order to observe and to try to better understand these types of events that Gustavo Sorá described as “complex events” (2004, p. 58). In recent years there have been critical advances from different areas of knowledge such as anthropology and ethnography (Sorá, 2013); history, literature and sociology (Bosshard & García Naharro, 2019; Villarino Pardo et al., 2021); and, more specifically, from the sociology of culture and literature, whose perspectives are adopted here.

In several of these studies there are proposals to define what IBFs are (Weidhaas, 2011, p. 201) and even attempts to classify them (Uribe Schroeder, 2012; Villarino Pardo, 2021) according to the perspective or professional field of the author and the moment in which they are published. According to some of these approaches, an international book fair (IBF) is a periodic event “in a concentrated space” (UNESCO, 2010, p. 119) that brings together the multiple actors of the book chain. At the same time, they are meetings that “provide an ideal opportunity for market research” (Owen, 2008, p. 103), in addition to being a space where “different kinds of values are produced, negotiated, and transacted” (Moeran & Strandgaard Pedersen, 2011, p. 2). They themselves configure a “ritual,” given that they are “extraordinary manifestations in relation to the rhythm of daily activity” (Sorá, 2004, p. 64).

In a special report of the International Publishers Association (IPA), *The future of Book Fairs* (2015) it is noted that “book fairs come in all shapes and sizes, attracting a variety of publics and serving different purposes” (IPA, 2015, p. 2). It is worth keeping in mind that IBFs have undergone important changes derived from new modes of circulation and transnational business (in particular with regards to the ebook and digital phenomena), of communication—especially linked to new technologies, of activities—opening up spaces to new concerns (sexual diversity, feminisms, functional diversity and disability, environment, bloggers, booktubers, bookstagrammers y *booktokers*^2^ etc.), and of celebration—fundamentally in the (post-) COVID-19 era.^3^

Thus, in recent years, we observe how in addition to the well-known models inaugurated by the *Frankfurter Buchmesse* and the *Salon du Livre de Paris*^4^ of inviting countries as honorees for each edition of these events or of celebrating “themes” (Bosshard & García Naharro, 2019; Galanes Santos, 2021; Muniz Júnior & Szpilbarg, 2016; Sorá, 2013; Weidhaas, 2011), there are several fairs—*Salon du Livre de Paris/Livre Paris*, the IBF of Buenos Aires and, in some specific case, the IBF of Guadalajara—that have opened an important exhibition space to present new *subjects* as guests of honor. It is, in a way, an instrument to rebalance positions in the transnational sphere as well as to perform other types of functions (Sorá, 2021, p. 43).

Thus, an explicit invitation is extended to:

- a culture: as is the case, for example, of “Catalan Culture” when it was awarded this distinction at the IBF Guadalajara 2004 and Frankfurt 2007;
- an autonomous community/region (and its culture): such as the cases of the Spanish communities of Andalusia (2006) and Castilla León (2010) at the Guadalajara IBF;
- to “themes,” entities or collectives of diverse nature: with the examples of “Latin America” at the Guadalajara IBF in 2016 or “Europe” at Livre Paris 2019^5^; and,
- specifically and more recently, to a city. This has been the stance, since 2011, of the Salon du Livre de Paris/Livre Paris (in parallel celebrating countries or other categories, until 2019); of the IBF of Guadalajara in its 2009 (Los Angeles) and 2017 (Madrid) editions; and, in a central way, of the IBF of Buenos Aires since 2013.

As we know, international book fairs are numerous and expensive (Owen, 2008, p. 106); therefore, each subject (individual or collective) or entity (public or private) must analyze and select the one(s) that best fit(s) its profile and possibilities according to the moment, and the objectives and strategies it can develop. Therefore, professionals in the book chain specialized in the sale and purchase of rights tend to opt for those in which the economic dimension of the book industry is a priority—without neglecting other capital at stake (symbolic, cultural, and literary)—and which have specific places for this purpose (Owen, 2008). Institutions (public and private) and policy makers must also make choices when deciding on the participation of a country, a culture/community, or a city in a fair (Dujovne & Sorá, 2010; Stella, 2020; Villarino Pardo, 2022) and, above all, if it includes the distinction of guest of honor given its interest in cultural promotion and internationalization actions (cultural and editorial). From the analysis of International Book Fairs—as a theoretical object or as an object of fieldwork—arises the hypothesis of considering how the IBFs themselves constitute, to a large extent and by themselves, types of *ephemeral/temporary cities* (Villarino Pardo, 2022, p. 186) that are are integrated into the dynamics of a city (Barcelona, Bogota, Bologna, Buenos Aires, Frankfurt, Guadalajara, London, Madrid, Paris, São Paulo, Shanghai, Sharjah, etc.).

We are in essence dealing with *Cities that, in turn, are hosted within cities* that they make visible in their names themselves (also as part of a *city brand*), and with which they dialogue through part of their cultural program. This is mainly visible in the case of the guests of honor (GoH) and when the program goes outside its fairgrounds and into the city that hosts it. And also, of primary importance, is the economic impact they generate on that city/region, linked to the book industry and, during the period of the fair, to various economic and tourism sectors. The latter aspects, for example, have been explicitly highlighted in the *I Informe económico sobre las dimensiones de la Feria internacional del libro de Buenos Aires* (First Economic Report Buenos Ares International Book Fair, 2020),^6^ commissioned by the Fundación El Libro (https://acortar.link/Xc30O7) in the last pre-COVID-19 edition. In the (post)pandemic period, they have also been evident in other cases: from the direct appeal to the host city made by the so-called “Special Edition” (2020) of the *Frankfurter Buchmesse* on its website^7^ to the impact experienced in the various sectors of these cities due to the cancellations of IBF in its face-to-face format in 2020 (https://acortar.link/GJDTuZ).

These events —mainly in their normal face-to-face versions—are a meeting place, at different levels, for the various agents of the book ecosystem given that, as the 2017 International Publishers Association (IPA) report recalled, “book fairs are a moment where many creative professions converge” (IPA, 2017, p. 3). In their in-person format the IBFs gather together (and it would be necessary to deeply analyze how the models that emerged in the hardest period of the COVID-19^8^ pandemic have been adapted) elements that recall the dynamics and spaces of a city (Villarino Pardo, 2022, p. 187), even in terms of their own forms of sociability (Vila-San Juan, 2007, p.103-110) derived from the face-to-face meeting “in the place”—in the case of professionals, after weeks/months of contacts and work, and “for the meeting”^9^—which includes parties at the venue. Thus, the editions following the toughest period of the pandemic by COVID-19, went from more restricted editions (in number of in-person participants, activities and volume, in 2021) to others that have been gradually recovering part of their previous numbers—as evidenced in the recent *Frankfurter Buchmesse* in 2022, whose participation was estimated to be 30% lower than in 2019 (Altares, 2022a).

## Cultural promotion and the status of guest of honor (GoH) at IBF

In recent decades, culture has been occupying a central role in the debates and concerns of specialists in increasingly broader fields, as well as in the programs and reports of numerous institutions of a national and international standing. In this paper we adopt a broad perspective of culture that includes, as the *Declaración de México* (1982) recalls, “in addition to the arts and letters, ways of life, fundamental human rights, value systems, traditions and beliefs” (UNESCO, 1982, p. 1). As Itamar Even-Zohar notes, in “the conception of culture as tools, culture is considered as a set of tools for the organization of life, at both the collective and individual level” (Even-Zohar, 2017, p. 77). And a document published by the Centro Regional para el Fomento del Libro en América Latina y el Caribe (the CERLALC, 2018), José Diego González points out that “international book fairs have become cultural activities of an undeniable benefit to citizens” (González, 2018, p. 9).

The visibility of countries, cultures or cities in certain IBFs—at least in models such as those of Frankfurt, Buenos Aires, Guadalajara, Liber Madrid/Barcelona or the *Salon du Livre de Paris/Livre Paris*—is associated with current strategies in the field of culture (and especially cultural policies^10^) geared towards fomenting and differentiating them at the international level. As we shall see, these are above all “branding” strategies that have become a tool of cultural diplomacy and *soft power* (as defined by Nye, 2008). It is worth bearing in mind, according to Morillo (2019, p. 3), that “today the brand is one of the most valued assets, not only by companies and organizations, but also by cities and countries.” Countries (and increasingly, cities) compete (beyond symbols or images) to achieve a *reputation*, to be *attractive,* i.e., “setting intangible values” (Morillo, 2019, p. 4), to differentiate among themselves and, among other things, to generate a pride of belonging among their inhabitants. At this point, we ask ourselves the following question: who is more interested in the distinction of guest of honor at an IBF, the fair itself or the honored subject? If we look, for example, at the case of the IBF of Buenos Aires, with more than four decades of tradition and a “mediatically baptized ‘festival of culture’” (Saferstein, 2013, p. 14), we see how, since 2013, it has incorporated the category of Guest City of Honor (with Amsterdam as the protagonist) into its model. On its website, when referring to the different honorees, it is explained that at the fair the organization intends to offer “special, rich, and distinctive cultural content and to help the initiation or strengthening of commercial relations between both book industries.”^11^ On the other hand, the authorities of some of the cities invited to the Argentine fair emphasize the ideas of “privilege,” “honor,” “opportunity,” “showcase/window,” also visible in other country case studies in relation to the meaning of a nomination of this sort. It also serves as a reference, and on this occasion for the Frankfurt IBF, the fact that Peter Weidhaas, the director between 1975 and 2000, recognized that “the Frankfurt Fair also benefited from these country presentations” (Weidhaas, 2011, p. 220).

Thus we know that the condition of GoH represents a cultural and economic showcase; it also requires planning—and not only economically, in the promotion of authorships, editors, translators, mediators and a cultural program (Galanes Santos, 2021; Muniz Júnior & Szpilbarg, 2016; Villarino Pardo, 2022) that benefit not only those who participate but also, as noted, the fair itself. This opportunity and what it implies to accept the commitment^12^ of starring in an event such as an international book fair (according to their respective models) in terms of planning and investments for years (GoH pavilion/stand, invited delegations, cultural program, curatorships, catalogs, programs to support the translation and circulation of authorship, etc.) for a country(/culture/city) guest of honor at that event were evident when observing what happened during the most complicated period of the COVID-19 pandemic.

Havana, chosen as guest of honor at the 45th edition of the IBF in Buenos Aires (2019), had to wait until May 2022 to make its real landing in the Argentine capital,^13^ already outside the time frame of the festivities for the 500th anniversary of its foundation. The case of Canada and the other countries that held that distinction at the Frankfurt fair in 2020 and the following years are particularly noteworthy.

We pause in this process related to the *Frankfurter Buchmesse* in order to observe how, due to its centrality on the international book fair circuit,^14^ the change in the model for holding the IBF in 2020 due to the COVID-19 pandemic, from face-to-face to mainly virtual, affected the entire book chain sector and who would occupy the distinction of GoH in the fairs adopting this model. In this scenario, Canada asked the organizers of the *Frankfurter Buchmesse* to postpone its presence as GoH in an edition that looked very uncertain in the months leading up to October 2020. Spain, Slovenia, and Italy accepted the fair’s invitation to postpone their participations by one year and that in the 2020 “Special Edition” the Canadian attendance would be smaller and in a primarily digital format. Thus, what was to be Canada’s first participation in the German event was twofold:^15^ a reduced telematic presence in 2020^16^ and a partly “conventional” way (retinue of guests, cultural program inside and outside the fair, etc.) in 2021,^17^ although—due to restrictions stemming from the pandemic—with a smaller number of participants and public.^18^ Despite the circumstances, the Canadian organization made a positive assessment of its participation.^19^

These movements promoted by Canada to achieve a greater impact through presence, given the exceptional conditions of 2020, confirm the importance of (continued) strategies of cultural policies and economic investment (public and private) not only for the internationalization of a country’s publishing market, as occurred directly in this case, but also for its cultural promotion and brand image (cultural diplomacy and soft power) through the participation as GoH in an IBF—especially in Frankfurt.

Cultural anthropologist Gustavo Sorá draws attention to the fact that “the focal themes and the countries invited as guests of honor objectify the core of the political component that makes up the internationalization of IBFs from non-English-speaking markets” (Sorá, 2021, p. 43). That political impact on IBF (especially linked to the GoH) is palpable in different ways. Most visible is the institutional representation at the opening ceremonies of these types of events, which have also become a political stageat a national and international level. To demonstrate this, we cite some examples linked especially to the German fair itself and to the cases we are dealing with here. Therefore, let us move on from the double Canadian participation in the aforementioned editions, to when Brazil (the first country to be given that distinction) was the GoH at the *Frankfurter Buchmesse* in 2013. The event was not attended by the President of the Republic, Dilma Rousseff, but by her Vice President (Michel Temer) and other members of the government and the country’s cultural institutions (*Ministério da Cultura, Academia Brasileira de Letras*, *Fundação Biblioteca Nacional*) in addition to the members of the GoH delegation and, on the German side, its Foreign Minister and the Director of the fair, among others. In the case of Spain 2022, the opening ceremony was attended not only by Ministers, authorities and entourage, but also by leaders at the highest level, with the presence of King Felipe VI and Queen Letizia of Spain and the German President Frank-Walter Steinmeier and some Ministers. It was a ceremony in which the Russian invasion of Ukraine was addressed in several speeches.

Thus, the *Frankfurter Buchmesse* 2022 edition will also be remembered more for the war in Ukraine and, in that scenario, for the intervention (videoconference) of Ukrainian President Volodymyr Zelensky (19/10/2022, https://acortar.link/6a6yso) than for the absence of Russian political and editorial representation. In fact, and for other reasons, Russia was also a political protagonist at *Livre Paris* 2018 when French President Emmanuel Macron, with Russia featured as guest of honor, issued a statement that the President of the French Republic had decided “in light of the international context, to not appear in the Russian salon, in solidarity with our British friends.” However, the President spoke with Russian intellectuals, authors and translators (https://acortar.link/Uud8qu). The French press referred to this fact in that inaugural session as “boycotting the Russian pavilion,” “ignoring the Russian pavilion,” etc.^20^ Part of the Russian delegation—and not only them—complained about this attitude and expressed their discomfort, commenting that such actions also had important economic repercussions among companies in the sector.^21^

Once again, it becomes clear that the IBFs—even in a (post)pandemic period—are venues for commercial negotiations in the publishing sector but also as outstanding instruments (/tools, in the sense of Even-Zohar, 2017, pp. 76-85) for cultural diplomacy and *soft power* (Villarino Pardo, 2014) and not only as a place for showing off authors or literary works (Machado de Assis, el *Quijote*, etc) as goods in a national/local cultural heritage sense.

This explains, among other reasons, the focus of certain institutions and local, regional, or state government authorities on cultural policy strategies in order to obtain this distinction by way of the participation of a GoH in an IBF. To this end, and at a moment of special visibility, the country (/city/culture) makes use of a visible accumulation of *energy*, as Even-Zohar calls it (2017, p.163, 183), and those fairs are selected which, for a variety of reasons, are considered more relevant for cultural projection (via cultural diplomacy and soft power) and for its publishing industry. These investments are not usually for a single isolated event, but cover multiple fairs, so that a certain “IBF circuit” (Frankfurt, Guadalajara, London, Buenos Aires, etc.) is formed in which, after a specific period of years, the subject’s GoH moment arrives.

As an example of this, we focus on the cases of Brazil and Spain, two countries that, in recent years, have opted for cultural diplomacy strategies through the condition of guest of honor at the main IBFs and because they pertain to linguistic systems that occupy peripheral (Portuguese) and semi-peripheral (Spain) positions, in what Heilbron and Sapiro (2008, p. 29) call the Global Translation System.

In both cases, different ways of presenting the same country to the world can be observed, and not only in the literary and publishing fields, as shown by the slogans they used at the most recent *Frankfurter Buchmesse*: “Brasil: um país cheio de vozes” (/“Brazil: a country full of voices”, 2013) and “España: Creatividad desbordante” (/“Spain: Overflowing Creativity”, 2022).

### Case studies: Brazil and Spain

After leaving behind a period of more than two decades of dictatorship, in the 1990s Brazil was a guest of honor at the following events (Table 1):

**Table 1:** Presence of Brazil as GoH at IBF (/Salon) in the 1990s

Brazil	1994	1995	1997	1998	1998
IBF(/Salon)	Frankfurt	Bogotá	Liber Madrid	Liber Barcelona	*Salon du Livre de Paris*

In 2005, as one of the results of the international country marketing *Plano Aquarela*, Brazil created a successful country brand. It was understood that, in addition to the image that a country represents by itself (Mariuti & Giraldi, 2012, p. 69), betting on a global marketing strategy benefited the exporting position (Kleppe et al., 2002) of its different strategic sectors (especially in tourism), in the current processes of globalization and transnational circulation of goods (also those of a symbolic nature) and in a scenario of international competitiveness.

Branding studies (country branding, city branding) represent an expanding area in the last thirty years (Anholt, 2000; Aronczyk 2013; Kaneva, 2011; Montanari et al., 2019) as well as their applications to countries and cities.^22^ In essence, it alludes to the “perceptions possessed by countries’ direct, indirect, real and potential consumers” (Valls, 1992, p. 28). Mariutti and Giraldi (2012, p. 70) also highlight that “the importance of the country in the global community, taking into account the political international economic, is a dimension of a country’s image,” among others. So, especially between 2005 and 2016, country branding strategies were understood by Brazilian leaders, during the first two terms of Lula da Silva and those of Dilma Rousseff (Rubim, 2010, 2015), as tools that implied an augment in the international arena—in terms of both symbolic and economic capital, for their products, institutions, companies, or actions.

Thus, the South American country, which has become a world economic power and with outstanding international projection^23^ at the turn of the first to the second decade of this century, showed its interest in translating this visibility into other fields (sports, cultural, publishing, etc.) and in taking advantage of certain events with significant media repercussions (Grix et al., 2015; Leão & Dennison, 2017).

So, in addition to the organization of the Confederations Cup (2013), the World Youth Day (Rio de Janeiro, 2013), the 2014 FIFA World Cup and the Rio 2016 Olympic Games; in the literary and publishing sphere, these strategies sought to project *the (contemporary) image of the country*, encourage the export of Brazilian literary and publishing production and consolidate its publishing market worldwide. To this end, different actions were designed and set in motion until the forced departure of Dilma Rousseff from government in 2016. These included direct support for Brazil’s participation as a guest of honor at major international book fairs, the renewal (2011) of the program (created in 1991) to support the translation of works and the dissemination of Brazilian authorship abroad (through the *Fundação Biblioteca Nacional*, in cooperation with the Ministério das Relações Exteriores do Brasil),^24^ the creation of the *Centro Internacional do Livro/Book Center Brazil* (2012), the publication of the *Machado de Assis Magazine. Brazilian Literature in Translation/Literatura Brasileira em Tradução* and, centrally, the commitment to the Brazilian Publishers project. This program, in place since 2008 under a successful agreement between the *Câmara Brasileira do Livro (CBL)* and the *Agência Brasileira de promoção de Exportações e Investimentos - ApexBrasil*,^25^ aims at promoting exports of the Brazilian publishing sector and its circulation in the transnational market, in addition to planning the sale of copyrights, organizing participation in IBF, and other related events and researching prospective markets. In this sense, the project also seeks to contribute “to the image and positioning of the country” (https://acortar.link/j6tRVI).

Precisely with this objective of showing the new economic, social, and political moment in the current century as well as the image of a modern country—far from the more traditional one it had shown in the tributes at the fairs of the 1990s—Brazil once again held this distinction at the IBF of^26^ (Table 2):

**Table 2:** Brazil as GoH at IBFs (/Salon) between 2000 and 2015

Brazil	2001	2005	2012	2013	2014^a^	2014	2015^b^
IBF(/Salon)	Guadalajara	La Habana	Bogotá	Frankfurt	Bologna	Göteburg	*Salon du Livre de Paris*

^a^São Paulo was the Guest of Honor at IBF Buenos Aires in 2014. The Brazilian metropolis is unique in the Brazilian publishing market, as shown by the “Publishing Production” data from the *Agência Brasileira de ISBN*. In fact, the State of São Paulo is home to the largest number of publishers per state in Brazil, as shown by the “Produção Editorial data” from the *Agência Brasileira de ISBN*.Vid. http://www.isbn.bn.br/website/relatorio-por-pessoa-juridica and http://www.isbn.bn.br/website/relatorio-por-pessoa-fisica.

^b^In 2012, when the program to support the internationalization of Brazilian literature was presented, with the new translation aid program, the plan envisaged a project until 2020, which also included participation as GoH in 2015 in the IBF in London and in 2017 in New York, which never occurred due to the drastic political changes in the country. *Vid.* https://acortar.link/KvEyRh

As Stella (2020, p. 162) pointed out, “in the 1990s, 2000s and 2010s Brazil attracted the attention of the international literary circuit.” What this international projection implies—and not only in terms of the accumulation of literary or economic capital in the publishing field—has been made clear on different occasions (Dujovne, 2022; Lindoso, 2011; Muniz Júnior & Szpilbarg, 2016; Stella, 2020; Villarino Pardo, 2014). As an example, the president of the *Fundação Biblioteca Nacional* at the time when Brazil was the GoH at the IBF Frankfurt 2013, Renato Lessa highlighted, after the opening ceremony, the fact that the fair was “an opportunity to promote the Brazilian culture” (Frey, 2013), and took the opportunity to promote the continuity of public policies and the support of the private sector for the internationalization of the Brazilian publishing market and literature.

In the case of Spain, the Proyecto de Country Branding was adopted at the end of 2001 with the aim of building, among several institutions “a new image that not only improves the economic projection of the country, but also transmits the new political, social and cultural reality, with its characteristics of modernity, artistic creation, dynamism and economic and cultural power” (Iglesias & Molina, 2008, p.115). According to the Global Soft Power Index of the consultancy Brand Finance in 2022—the most recent of this nature—Spain increases its power of influence and is placed, according to its metrics, at 11th place, with the Culture & Heritage field (5th place) being one of the best valued (https://acortar.link/zHivkf). The 2019 publication of *The Soft Power 30* by Portland Consulting already highlighted the importance of Spain’s cultural legacy in its soft power (https://softpower30.com/country/spain/).

In the concrete political and cultural scenario of recent years, Spain^27^ has again taken an interest in IBF presence as a way to expand its international projection through literature and—in the broad sense that we adopt here—culture, as have other surrounding countries distinguished with the status of guests of honor of the *Franfurter Buchmesse* (in this period, France in 1989 and 2017 or Italy, in 1988 and 2024), This is shown by the fact that it has been honored at the following international book fairs in recent years (Table 3):

**Table 3:** Presence of Spain as GoH in IBF between 2019 and 2025

Spain	2019	2021	2022	2022	2023	2024	2025
IBF	White House (SIEL)	United Arab Emirates (Sharjah)	Frankfurt	Slovenia	Calcutta	Guadalajara	Bogota

The capitalization that Spain intends to make of these current investments in transnational cultural projection is shown, in part, in the institutional presentation of the agreements that allow this cultural and literary mission and in the significant fact of framing these actions in March 2022 as reinforcement of “the mission and investments that Spain is making through the PERTE of the new economy of language” 28(https://acortar.link/AabyPn), with primary funding coming out of the *Plan de Recuperación, Transformación y Resiliencia* (“*The Recovery, Transformation and Resilience Plan*”) of the European Union (PERTE).

These actions are complemented by the boost given to the translation and sale of rights of Spanish works (two of the main objectives of this internationalization program),^29^ in the country’s different official languages, on the *Books from Spain* website, an initiative of the Ministry of Culture and Sports (MCyD), *Acción Cultural Española* (AC /E) and the *Federación de Gremios de Editores de España* (FGEE), created in July 2020 with the aim of promoting the sale of rights—especially online during the period in which there were no face-to-face professional meetings due to the pandemic^30^—and making the most of Spain’s participation in the German fair, but not only at the IBF (https: //short. link/1cX51y). An objective that, according to the organization—as well as in the opinion of several authors—and at the end of the fair, would have been satisfactorily achieved^31^ both in terms of the impact during the days of the IBF and in the volume of translated works and, consequently, in the Spanish publishing industry’s process of internationalization.

The organizers of Spain GoH at the *Frankfurter Buchmesse* insisted (and it was echoed in certain media) that it was a “State project” (https://acortar.link/fVsduQ). A state cultural policy plan in whose discourse actions such as the Spanish participation^32^ as guest of honor at the Sharjah IBF in 2021 have contributed/contribute, where it was emphasized that this protagonism is a (prominent) action of a broader strategy of projection. In this case it was explained, in a place “Where, for several years now, cultural diplomacy has been working to turn Spanish creations into star brands as well as attracting high-end tourism” (Carroto, 2021).^33^

Spain was presented as GoH at the 2022 *Frankfurter Buchmesse,* being the 5th country in production of book titles —both in paper and digital format— following the United Kingdom, the Russian Federation, France and Italy 34and with a publishing market very different in nature to that of 1992, one which “has undergone a great transformation to become the main cultural activity in our country” (FGEE, 2021, p. 9). According to the 29th study of the Foreign Book Trade prepared by the Association of Spanish Chambers of Books (ACLE), “The publishing industry is one of the most internationalized sectors of the Spanish economy in intensity and longevity” (FGEE, 2021, p. 8). Regarding rights, the aforementioned report pointed out that in 2020, a total of 72.89 million euros was invoiced in rights sales, an increase of practically 2 million euros, “English being the language in which the most rights are sold” (*ibidem*). The same document explains that “efforts to consolidate and increase these new markets are still necessary.”^35^ That current figure falls a bit from the 63.01 million euros of rights sales in 2009 and remains in slightly better figures than those of the last 5 years (2015–2020) (ACLE, 2021, p. 33). We will have to wait to know the specific figures relating to 2022 and the possible Frankfurt 2022 effect.

Here again, *SpainFrankfurt* 2022 was intended to constitute a medium-term cultural policy strategy. To this end, it is also emphasized that the translation aid program initiated in 1984 is “a basic tool” and that, with this project for 2022, its budget was significantly increased and it gained greater visibility in terms of the number of titles, publishers, and authors benefiting from the program. The press dossier of the Spain Guest of Honour Frankfurt 2022 Program stresses that these support initiatives “have proven to be an effective instrument for the international dissemination of our literature in all the languages of the State,” so, for the occasion, Acción Cultural Española (AC/E) added a specific program, “this time focusing on five strategic markets defined with the sector: German, English, French, Italian and Dutch.” (https://acortar.link/BiyIBt). A choice that, as we shall see, takes into account the weight of these languages in international translation flows.

Thus, still pending the resolution of the 4th edition (https://acortar.link/MgmaPK), the “Radiography of grants for the translation of literary and scientific works written in Spanish into foreign languages” (https://acortar.link/1NTL4q) shows that this aid program of the MCyD of the Spanish government and AC/E, between 2019 and 2022, has allocated a total of €3,160,000^36^ to the promotion of translation into foreign languages. It would have benefited 1313 applications from “Spanish publishers and agents for the translation of fragments (copyright sales dossiers)” and of “foreign publishers for the translation of complete works, anthologies and support for illustration.” (*ibidem*).

**Fig. 1:**
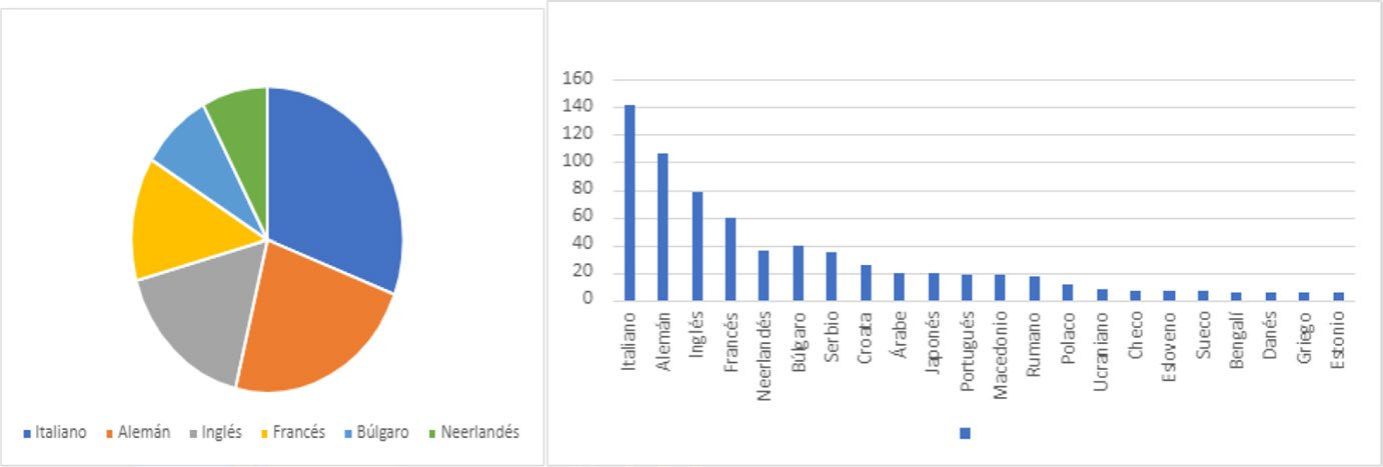
Our elaboration based on data from SpainFrankfurt 2022 (https://acortar.link/1NTL4q)

In this balance sheet, the strategic markets for the translation of Spanish titles, according to the number of works translated during that period,^37^ are as in (Figure 1).

These are strategies adopted by states to support cultural exchanges at the transnational level with a view towards reorienting export policies and responsibility for tasks traditionally associated with the diplomatic sphere (Sapiro, 2008, p.12). This is intended to favor the transnational circulation of Spanish works and authorship and, specifically, the sale of title rights to markets beyond Germany.

To better understand this data, both in the analyses of the Spanish and Brazilian cases, it is worth bearing in mind that transnational cultural exchanges, as Heilbron (2009, p. 259) reminds us, have their own dynamics. Thus, as in other aspects of the circulation of symbolic goods, these transnational flows reveal logics that are not necessarily in direct correspondence with the geopolitical positions of countries or with the number of speakers of a language. Jean Heilbron stresses that “the international translation system is above all a hierarchical structure with central, semi-peripheral and peripheral language groups.” (Heilbron, 2009, p. 261). A system in which, as we noted, English—and not only for the period initially studied—occupies a position of hypercentrality, followed by French and German (central positions) and other languages in semi-peripheral positions (Spanish, Italian, Russian and Swedish) and peripheral (Arabic, Chinese, Portuguese, etc.) ones (Es & Heilbron, 2015, p. 3). In that framework, the usual translation flows go from center to the periphery rather than in the other direction (Heilbron & Sapiro, 2008, p. 29), in a movement in which “communication between the peripheral groups often takes place through a center” (Heilbron, 2009, p. 264).

Given that the main objective is the transnational circulation of this symbolic good, those linguistic spaces or publishing markets that can function as springboards or as channels of intermediation between linguistic-cultural-publishing spaces are usually seen as important for those who plan or manage these types of dynamics—at the private or public level. So, the main languages into which Spanish works have been translated on this occasion including the different languages of the State) with the support of these programs during the period analyzed (2019–2022), are target languages or springboards to another—with English as a prominent destination or “object of desire.”

Returning to the case of Brazil, and on the occasion of the “30-Year Review of the Brazilian National Library Foundation’s Translation Program (1991–2021),” the Book Center Brazil published a balance of the program on 7/20/2021 in which it was highlighted that, up to that moment, and during those three decades, there was a total of 1,114 translations produced by the program, corresponding to 350 authors. Looking at the data of the last decade (2010–2019), the figures reveal a greater international interest in the translation of Brazilian works, distributed as follows: “fiction books received 658 translation grants, followed by non-fiction (132), poetry (73), children and youth literature (58), comic books (29) and theater (9)” (https://centrointernacionaldolivro.wordpress.com/).

**Fig. 2:**
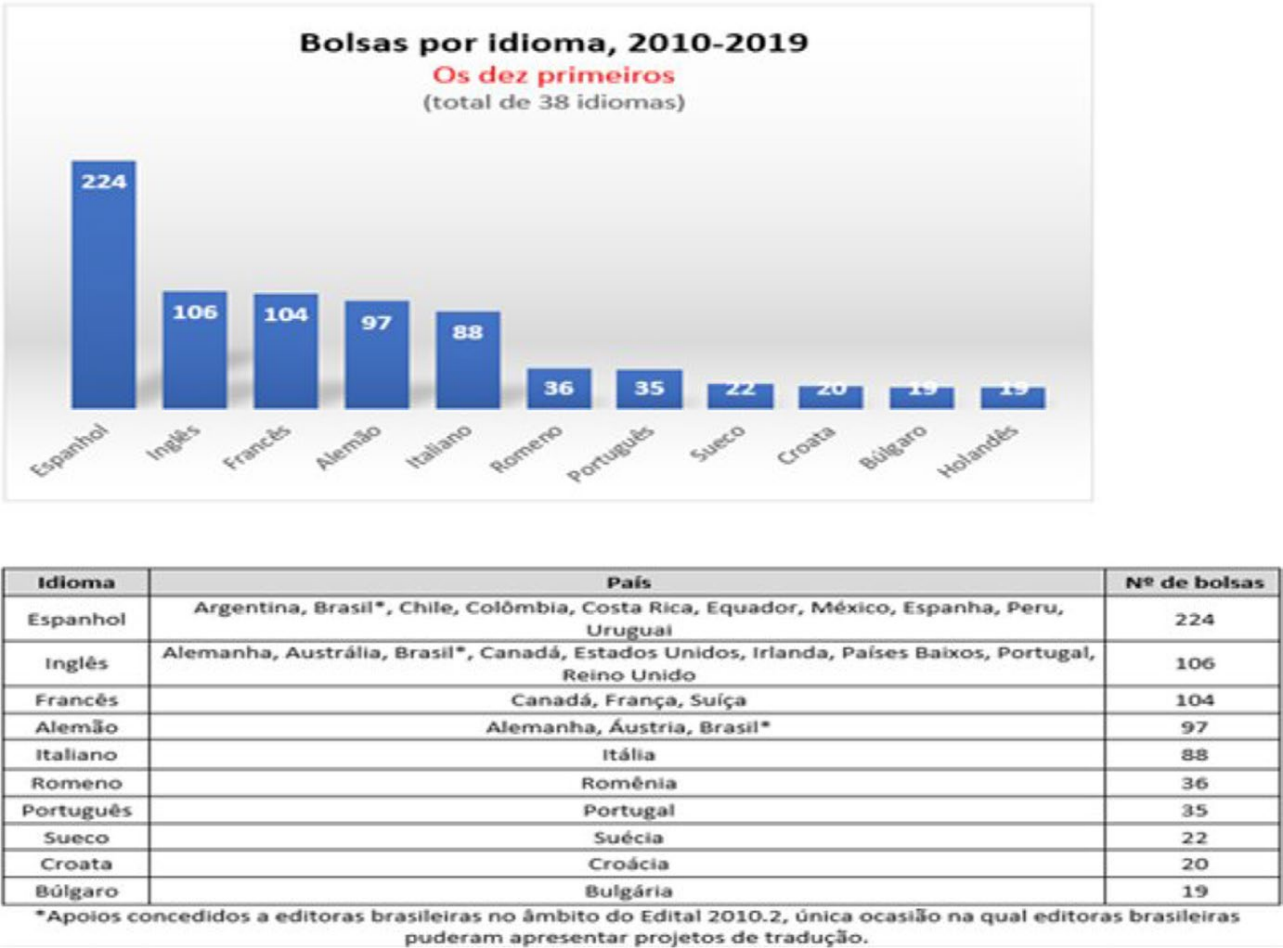
Results of languages benefited by the translation program (2010–2019). *Source of graphs*: https://acortar.link/ZiK58P

Over the past decade, these translations have been translated into the following languages and countries (Figure 2).

**Fig. 3:**
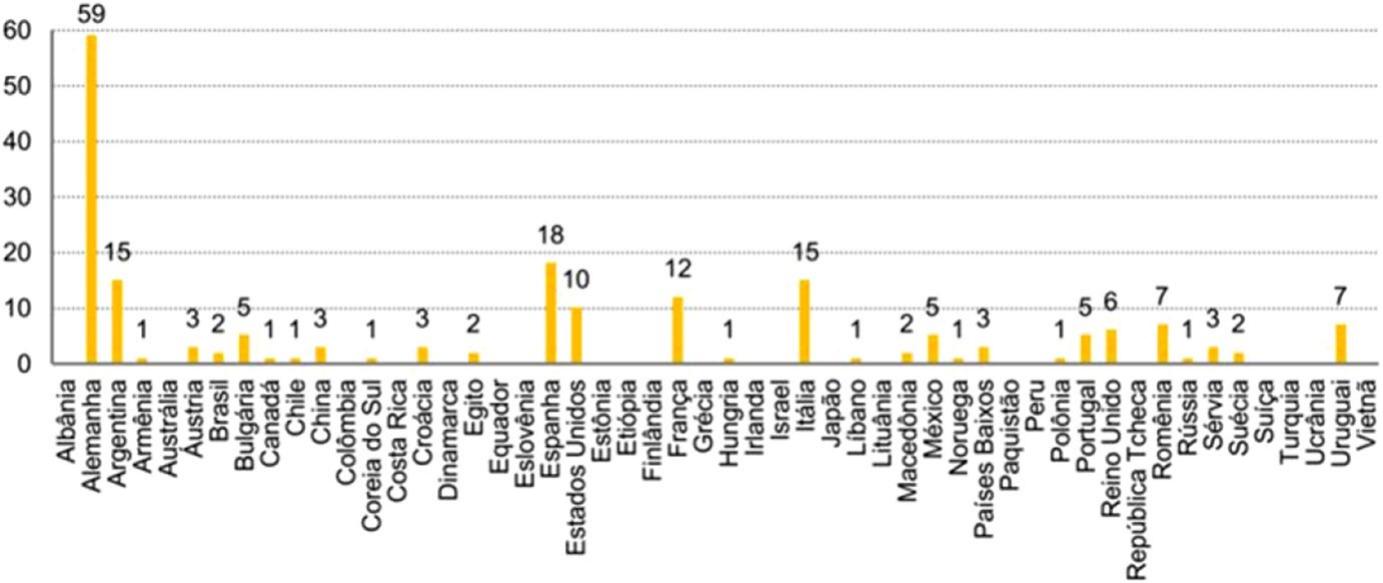
Feres and Brisolara (2018, p. 339)

**Fig. 4:**
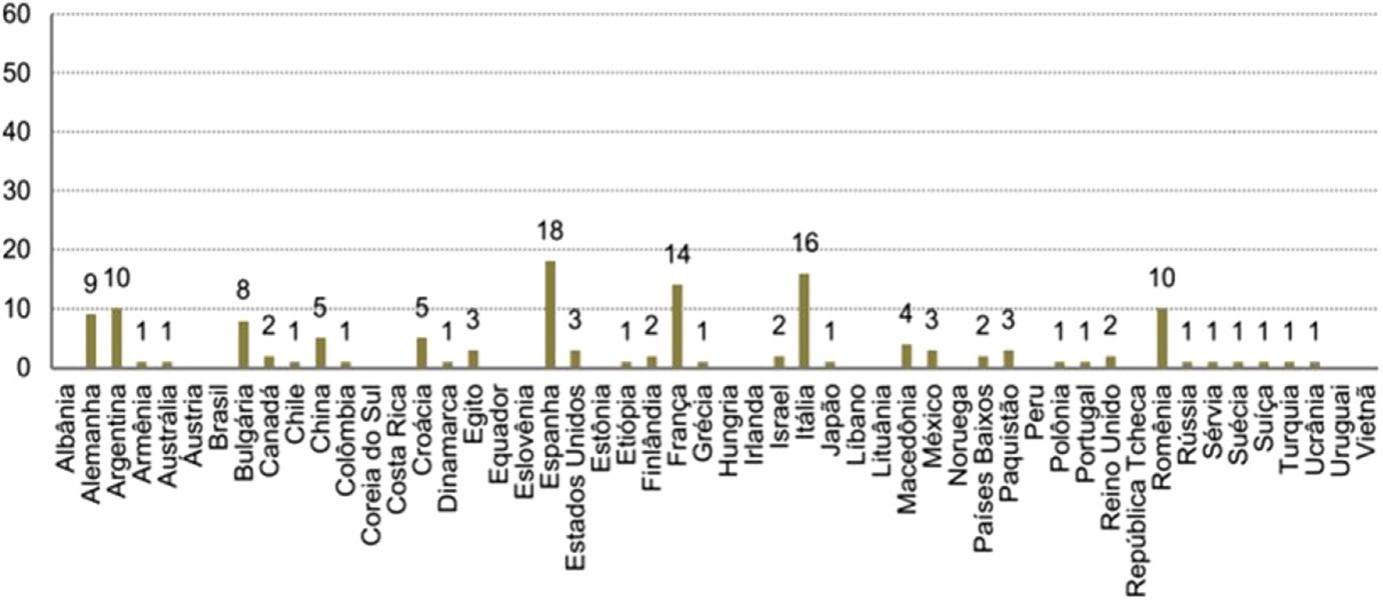
Feres and Brisolara (2018, p. 339)

If we take a closer look at that period—as Feres and Brisolara (2018) do—we note how the renewal of the Program to Support the Translation and Publication of Brazilian Authors Abroad in 2011^38^—just when the country was preparing for its premiere as Guest of Honor at the *Frankfurter Buchmesse*—reveals a notorious increase of “1,080%” of translations in Germany in the 2013 call (59 works) in relation to the volume of translations in the previous year (5) (Feres & Brisolara, 2018, p. 339); in a context of an increasing number of translations, countries and languages, as shown by these authors in the following graph (Figures 3, 4).

In the following years and until the last IBF in which Brazil was honored as the Guest of Honor Country (*Salon du Livre de Paris*, in 2015), the transnational interest in the translation of Brazilian literary works, to a large extent (as we observe from the data of the translation program) moves according to fair appearances. Thus, in 2014 and according to the same authors, there were 136 works translated and published in 30 countries; France being the country with the largest number of titles stemming from the program—“25 títulos (contra 12 do ano anterior)” (Feres & Brisolara, 2018, p. 340). In contrast to this increase in the *Salon’s* host country, in the year following the Brazilian presence at the *Frankfurter Buchmesse* in 2013 when its publishers garnered 59 published titles under the program, they mention that “surprisingly,” “Brazilian works are simply not published in 2014, either because no German publisher applied for the grants or because none of those who applied had their project selected to receive the incentive” (Feres & Brisolara, 2018, p. 340). Spain (the country that has benefited most from this type of aid) and Italy continue to be countries with an interest in translating Brazilian works throughout those 30 years and in the specific period of 2010–2019 (focus of the *Book Center Brazil* analysis).

As published in 2005 in one of the periodic bulletins of the Vivaleitura program^39^—linked to the *Año Iberoamericano de la Lectura*—at a time when Brazil was guest of honor at the IBF in Havana^40^ and was the “eighth largest book producer in the world and the largest publishing industry in Latin America” (Assessoria Vivaleitura, 2005), in this (necessary) process of internationalization, not only would the different members of the book production chain benefit, but “in the export of publishing products to the entire culture in international projection” (*ibidem*).

## Final considerations

Currently, international book fairs (and, increasingly, literary festivals) have a marked calendar not only in the annual agendas of the various actors involved in the publishing market but also of those responsible for cultural policies at various levels. Through their different models, IBFs have become increasingly important places for the mediation and attainment of capital (symbolic, economic, literary or cultural) and positions in a supposedly transnational literary (cultural and publishing) field, not only for publishers, literary agencies or authors, but also for countries, cultures, and cities. Thus, in addition to those bodies representing authors,’ publishers’ or translators’ guilds, as we have seen in different cases, there is also a real interest shown by various leaders and government bodies (at local, regional, state or supranational level) that include the participation of countries/cultures/cities in these events—particularly via the condition of guest of honor—as strategic attempts to publicize certain aspects of branding (country brand, city brand).

In instances such as the IBFs, these participants, especially in the condition of GoH, stage a representation of ‘the national’/’the local’/’the common’ (depending on the type of participant), of how they wish to be seen and (re)known at that moment. Through a program (literary and cultural) and visual representation (also in the presence of the fair and the city that hosts it) they take advantage of the fact that the event attracts significant media and public attention and present their ‘story(s)’—those that, to a large extent, define and summarize them at that moment. In short, a way of conveying their image (country/city/culture). This is what, at different times and in different IBFs, countries such as Brazil or Spain have done. Their participations in the *Frankfurter Buchmesse* of 2013 and 2022, respectively, showed those “stories” that they summarized in their slogans, based on several pillars: In the South American country’s presence *antropofagia* is the central theme consisting of six thematic emphases (a country full of voices, in permanent cultural co-creation; plurality; cosmopolitanism; diversity; miscegenation; bibliodiversity). In the Spanish case five foci are present (bibliodiversity, innovation, linguistic plurality, gender equality, and sustainability).

It is clear that IBFs are cultural events that are also political events. Public institutions—at different levels—and private institutions—generally publishing groups—participate in these dynamics as organizers, aware that they must take advantage of the opportunity to make the most of these mechanisms of internationalization (of a literary system and a publishing market, especially through translation and the sale of rights) and the acquisition of capital in order to present a certain country image as well. What happened with Canadian participation at the German fair (and the subsequent ones—including the Spanish—during the COVID-19 pandemic) is a good example of this. In fact, the Canadian organization explained that being GoH at the *Frankfurter Buchmesse* meant “a prestigious opportunity to showcase a country’s publishing, cultural and creative industries. It is an event of international significance akin to the World Expo and the Olympics for literature. On the international publishing stage, no other event offers a comparable level of visibility and impact” (https://canadafbm2021.com/).

Hence, the IBFs are now opportunities and *tools* for promoting not only literature and culture but also tourism and creative industries (most of all in the publishing sector), and for making the position of these social and territorial entities visible in a global scenario of symbolic goods by means of their literary and cultural projection at these events. So, by way of an open-ended conclusion, the question arises: once you have completed a certain IBF circuit as a guest of honor on more than one occasion—being invited twice at the *Frankfurter Buchmesse*, for example, like Brazil or Spain—where is the next stop towards achieving the goals that you fulfilled there?

## Footnotes

1 Moeran & Strandgaard Pedersen (2011, p. 2) recall that these meetings have been, until a few years ago, widely ignored in the academic field (especially in fields such as economics, anthropology, sociology and management).

2 As a sign of their ability to influence certain consumers, the last edition of the Frankfurter Buchmesse (October 2022) teamed up with Tik Tok (where the #BookTok hashtag is increasingly present) as one of its partners.( https://acortar.link/ZFo0lv. link/ZFo0lv); and, in April of the same year, the IBF of Buenos Aires updated the name of its “International Booktubers Meeting” to “International Bookfluencers Meeting” to adapt to the different social networks in which, especially young audiences, approach and recommend books (https://acortar.link/BwM7Wz).

3 In 2020 and after the pandemic was declared for COVID-19, the different organizations of the main IDF decided, by a majority, to cancel their respective face-to-face editions and move their celebrations to the digital environment, through their websites. Several examples of proposals to resolve the situation have been analyzed by Sabogal (2020) who, in October 2020, summarized the ordeal experienced by the different IDF as follows: “A swinging door between the physical and virtual worlds [...] They have been undergoing a metamorphosis on the fly for the last six months.”

4 A format that has been transformed, not only in name—Livre Paris since 2016—but also in the event itself. In 2022, and after two years of cancellations, it returned with a different formula and emphasis: *Festival du livre de Paris* (https://www.festivaldulivredeparis.fr/).

5 And, in 2023, at the Guadalajara IBF as well.

6 The Report details the expenses derived from the organization of the Argentine fair—in the 2019 edition—and points out that “The main economic beneficiary of these items is the City of Buenos Aires itself.” He also stresses that, with the model of holding the IBF up to that moment: “There is also a beneficial international impact for the whole country, since when a fair is placed in a prominent and prestigious way on the world calendar, the national identity image is reinforced. And it has an intangible result, difficult to measure” (https://acortar.link/Xc30O7).

7 “All together now—On air. At the fair. In the city” (https://acortar.link/xNmo5f (19/11/2020).

8 For example, the cancellations of the Buenos Aires IBF editions in 2020 and 2021, for example, have led to a greater presence of digital content on its website (https://www.el-libro.org.ar/). In the case of the Guadalajara IBF, for the first time in its history, a “special online edition” was chosen (https://www.fil.com.mx/invitado/2020.asp). For the Frankfurter Buchmesse it involved, among other innovations, the creation of the Frankfurt Rights platform (https://frankfurtrights.com/-) in which the website *Books from Spain* was included.

9 After two years of absences due to the pandemic, one of the Spanish publishers (Luis Solano, from Libros del Asteroide) reflected on the matter and summarized, in part, a generalized opinion: “there is the uncertainty of whether it is worth it or not, [...]. But it is still very important to be here, there are editors with whom you meet once or twice a year and one of them is here. It’s something I miss and it’s interesting. There are many things that can occur over a coffee, in a hallway” (Altares, 2022b).

10 Regarding this issue for Ibero-America, see Dujovne (2022).

11 See https://www.el-libro.org.ar/internacional/ciudades-invitadas-de-honor/ (04/06/2022).

12 On the type of support given by the GoH for the Frankfurt IDF, see Sorá (2013, p. 104).

13 See https://acortar.link/E5VXQj; https://acortar.link/rzRldO.

14 Considered “the key event in the trade fair calendar” (Owen, 2008, p. 106); “the epicenter of the publishing business that it is today” (Uribe Schroeder, 2012, p. 21); ‘the most relevant worldwide’ for the publishing market (Weidhaas, 2011); or the “most important in the world” (Sorá, 2004, p. 61).

15 See https://canadafbm2021.com/assets/FBM2020_%20Report_EN_MAR29.pdf

16 As samples of this modality we refer, for example, to the speech of the country’s Prime Minister, Justin Trudeau, on the occasion of this edition of the fair (https://twitter.com/i/status/1316063611308081153); or references in other media such as this: “This digital version of the Frankfurt Book Fair 2020 changed traditions; Canada, the special guest country will not be in person, but virtual” (https://acortar.link/9rUa87, 10/14/2020).

17 The striking slogan of the edition was: “Re:connect.”

18 For example, with speakers Margaret Atwood and Joséphine Bacon telematically delivering their respective speeches at the opening session *Vid.* https://canadafbm2021.com/virtual-pavilion/; https://acortar.link/J06AG.

19 *Vid.* https://canadafbm2021.com/; https://acortar.link/WhBywH.

20 *Vid.* https://acortar.link/sD6Hy8; https://acortar.link/Q1EcOG.

21 On complaints to that fact, see *Le Point* (18/03/2018: https://acortar.link/w4JvLd).

22 With methodologies and analysis applied by different companies and consulting firms. Some of the best known are: Anholt-GfK Roper Nation Brands Index (NBI) and FutureBrand Country Brand Index (CBI).

23 A summary of the different studies on the country’s international projection and position during that period and until 2022 can be found in Hochleitner (2022, pp. 169-184).

24 *Vid*. https://acortar.link/PBho7r; https://acortar.link/yirzwp.

25 *Vid*. http://www.brazilianpublishers.com.br/.

26 In addition to being honored in the 2011/2012 edition of the Europalia Festival (https://europalia.eu/fr/a-propos-deuropalia/archive).

27 Spain returned to the German IBF in 2022, after 31 years of having been GoH. It does so with a “journey” spanning the slogans “La hora de España,” with which the country was presented in 1991 on the eve of the Barcelona Olympics and the Seville Expo, and “España, creatividad desbordante,” its 2022 title, through the program composed by the platform “Spain Frankfurt 2022” (https://spainfrankfurt2022.com/es/).

28 A legislative and economic framework (with a budget of 1.1 billion euros in public investment and the goal of reaching an additional billion euros in private investment) that was presented at the *Frankfurter Buchmesse* by the First Vice President of the Spanish Government, Nadia Calviño (https://acortar.link/onsVh9). See https://acortar.link/YhqjvB.

29 *Vid*. https://acortar.link/BiyIBt.

30 Among others, the IBF of London, Bologna and, in part, Frankfurt (https://acortar.link/BiyIBt).

31 In fact, they refer to the project as “mission accomplished” (https://acortar.link/pCucsT), “objective reached” (https://acortar.link/fVsduQ), “a success story” (https://acortar.link/s9UfNf) or “‘overwhelming’ balance” (https://acortar.link/lT0eu0).

32 See https://acortar.link/6OzpY0; https://acortar.link/nxSoqC.

33 It represented, in the words of Culture Minister Miquel Iceta, an opportunity, a “cultural interest in ‘opening more doors to the East, and the door is Sharjah” (Carroto, 2021). The Sharjah IDFS (SIBF) 2021, and because of the pandemic circumstances, turned out to be “the world’s biggest book fair this year” (FGEE, 2021, p. 10).

34 See https://acortar.link/Sdjlhv based on data from the World Intellectual Property Indicators 2020 (https://acortar.link/xSuwY3).

35 Of these, in 2020, “22.5 million, or 31.70%, was translated into English” (in the UK, Australia, New Zealand, etc.). And, “the other languages have remained in the same order, although translation into French and German has decreased, while translation into Chinese and other languages has increased, although the evolution of the sale of rights is positive because it is growing” (ACLE, 2021, p. 33). According to the same source, these are, in this order: English, French, German, Italian, Dutch, Japanese, Chinese and other languages (ACLE, 2021, p. 34).

36 Distributed as follows: €660,000 in 2019, €800,000 in 2020, €850,000 in 2021 and €850,000 in 2022 (“Includes AC/E’s pending call for foreign publishers 2022”) (https://acortar.link/1NTL4q).

37 Regarding the SpainFrankfurt 2022 Program, it should be noted that: “In 2022, 135 Spanish titles have reached or will reach the German market, most of them translated from Spanish or other languages of the State” (https://acortar.link/1NTL4q).

38 In this regard, one can consult information from the *Book Center Brazil* (https://acortar.link/KvEyRh).

39 It was linked to *Ministério da Educação e da Cultura* (2005), but it has not been possible to access their website for some time: http://portal.mec.gov.br/component/tags/tag/vivaleituras

40 https://acortar.link/2KquEr.
